# Oral Surgery—Theory and Results

**Published:** 1897-05

**Authors:** G. Lenox Curtis

**Affiliations:** New York


### Oral Surgery—Theory and Results.

BY G. LENOX CURTIS, M.D., NEW YORK.

Abstract of an address read before the Canadian Med. Association, August, 1896.

Theories in surgery, as in finance or government when founded on insufficient data, are apt to be exploded when an attempt is made to demonstrate their real worth by practical test. For it is undeniable that results are the true criteria of the value of work. A theory which will not, at all times, bear this test fails.

When general surgery and many of its special branches have been brought to a high degree of perfection, where theory and result accord beautifully, there are departments of the great work of no less importance than the fields now cultivated by the medical profession which are utterly neglected in the teachings of the medical institutions and in the practice of medical men. The physician considers it beneath his dignity to investigate the mouth as an indicator or cause of disease further than to look at the tongue. He will not refer to the teeth lest he may be classed with the "dentists." Yet the mouth, which is the gateway to the alimentary tract, the portal through which passes the food which nourishes the body, would seem to demand his first and closest consideration.

The completeness of the lack of knowledge on the part of the average physician and surgeon concerning diseases attendant upon or following affections of the teeth, of the effects, near and remote, which such affections may cause in the organism is appalling. Many times their patients suffer untold agony or endure prolonged illness because of the physician's ignorance upon these subjects, which should be among the fundamentals. For much, if not all of this, the medical institutions of learning are responsible. In the curricula of many of these the teeth, in spite of all the attention that is given to them and their diseases, let alone their anatomical and nervous relations to the remainder of the economy, might well be foreign bodies.

There are many cases where the dentist discovers the cause of the trouble with the patient's general health, but he is overruled by the physician, whose authority and knowledge are supposed, by the patient, to be supreme.

Few, perhaps, have better opportunities than I to see the evils which flow from the physician's ignorance upon the subject of the teeth. The physician, in formulating his theories for the explanation of obscure troubles, entirely ignores this factor. He has never been taught to appreciate the teeth as a possible element in any disorder but toothache, or perhaps a neuralgia of the face. The medical schools are no aid to him, the text-books give him no inkling of the truth. The teeth are the province of the dentist, and the dentist is too often looked upon with contempt by his medical confrere as being a one-sided, semi-educated man, when really this very one-sidedness has made him a master in oral and facial diseases. Upon these points the dentist does not vainly theorize. He gets results, and these results are his recommendations to the medical profession.

It seems to me that in this day of enlightenment upon the teeth among dentists it is almost criminal in the medical institutions of learning to send their graduates out with less than half the knowledge that should be given them. If this be so with regard to colleges, what shall we say of the man, who, practicing the most beneficent profession in the world, fails to acquaint himself with a subject so important to the sound pursuit of medicine? Is he not lacking in his duty to himself and to his patients? With so important a factor in many cases entirely omitted, can he do more than vainly experiment upon his patients, blindly groping for what he has not eyes to see?

I have no objection to experimenting with patients with a view to further enlightenment, provided it is done honestly and with all the possible known elements estimated at their true value. But when the experiment is necessary because the physician or surgeon lacks common practical knowledge, which he can easily avail himself of, I can not uphold it. Such a course must necessarily be merely mercenary. A theory formed by such a man must be wrong, his practice can not help being mischievous, his results, so far as good is concerned, will be nil. He is simply a " guesser," and while he is guessing his patient's life may be slipping away.

It behooves us, then, to endeavor by every manly means to free ourselves from every environing circumstance which tends to cramp our efforts to relieve human suffering. What we are after are results, not theory. As we can learn industry from the little busy bee, or patience and perseverance from the spider, so we may even learn from the dentists the relations of the teeth to apparently unrelated lesions. Certainly we should neglect no source of information which would strengthen or enlarge our means of fighting disease. So, and so only, shall we be able to confer upon our patients the highest benefits within the limits of our profession.—*Am. Medico-Surg. Bulletin.*

